# The influence of persistent organic pollutants in the traditional Inuit diet on markers of inflammation

**DOI:** 10.1371/journal.pone.0177781

**Published:** 2017-05-19

**Authors:** L. K. Schæbel, E. C. Bonefeld-Jørgensen, H. Vestergaard, S. Andersen

**Affiliations:** 1 Centre for Arctic Health, Department of Public Health, Aarhus University, Aarhus, Denmark; 2 Arctic Health Research Centre, Institute of Clinical Medicine, Aalborg University, Aalborg, Denmark; 3 The Novo Nordisk Foundation Center for Basic Metabolic Research, Section of Metabolic Genetics, Faculty of Health and Medical Sciences, University of Copenhagen, Copenhagen, Denmark; 4 Department of Geriatric and Internal Medicine, Aalborg University Hospital, Aalborg, Denmark; 5 Institute of Health Sciences, Ilisimatusarfik, University of Greenland, Nuuk, Greenland; Stony Brook University, Graduate Program in Public Health, UNITED STATES

## Abstract

Concentrations of persistent organic pollutants (POPs) are high in Inuit living predominately on the traditional marine diet. Adverse effects of POPs include disruption of the immune system and cardiovascular diseases that are frequent in Greenland Inuit. We aimed to assess the association between exposure to POPs from the marine diet and inflammation, taking into account other factors such as vitamin D. We invited Inuit and non-Inuit living in settlements or the town in rural East Greenland or in the capital city Nuuk. Participants completed a food frequency questionnaire and donated a blood sample for measurement of the two markers of inflammation YKL-40 and hsCRP, 25-hydroxy-vitamin D, eleven organochlorine pesticides (OCPs), fourteen polychlorinated biphenyls (PCBs), one polybrominated biphenyl, and nine polybrominated diphenyl ethers (PBDEs) adjusted to the serum lipid content. Participants were 50 through 69 years old, living in settlements, town or city (n = 151/173/211; 95% participation rate). ΣOCP, ΣPCB and ΣPBDE serum levels were higher in Inuit than in non-Inuit (p<0.001/ p<0.001/ p<0.001), in older individuals (p<0.001/p<0.001/p = 0.002) and in participants with the highest intake of Greenlandic food items (p<0.001/p<0.001/p<0.001). Both YKL-40 and hsCRP serum levels were higher in Inuit compared to non-Inuit (p<0.001/p = 0.001), and increased with age (p<0.001/p = 0.001) and with the intake of Greenlandic food items (p<0.001/p = 0.002). Multivariate analysis conformed to a marked influence on both YKL-40 and hsCRP by ΣOCP (p<0.001/p<0.001) and ΣPCBs (p<0.001/p = 0.001) after adjusting for age, BMI, vitamin D, alcohol and smoking. POP levels were associated with the intake of the traditional Inuit diet and with markers of inflammation. This supports a pro-inflammatory role of POPs to promote chronic diseases common to populations in Greenland. These data inform guidelines on ‘the Arctic dilemma’ and encourage follow-up on the ageing Arctic populations.

## Introduction

Persistent organic pollutants (POPs) are typically halogenated organic compounds derived from industrial activities [1]. They are classified based on chemical structure to include polychlorinated biphenyls (PCBs), organochlorine pesticides (OCPs) and polybrominated diphenyl ethers (PBDEs). They resist degradation remaining intact for many years. Hence, they are persistent [2].

The chemical properties cause POPs to be convenient for a number of purposes and their use commonly includes flame-retardants, lubricants and pesticides [1]. Once released into the environment they become widely distributed and long-range transport of POPs to the Arctic occurs by atmospheric and ocean currents [2–4].

POPs are highly lipophilic and bio-accumulate in fatty tissues of animals and humans. Hence, bioaccumulation occurs in addition to magnification in the food chain that cause high concentrations in top predators [1,3].

Due to long-range atmospheric transport human exposure to POPs is ubiquitous and not restricted to individuals living in industrial areas, large cities or parts of the world where POPs are still in use. Populations in the Arctic may even experience larger exposure to POPs than populations living in industrialized parts of the world.

Inhabitants in Arctic Greenland still rely on the traditional diet comprising of fish and marine mammals at the top of the marine food chain [4–6]. Hence, they are exposed to POPs largely due to their reliance on the traditional Greenlandic diet. Despite being banned or restricted for several years, concentrations of POPs are still high in marine mammals in Greenland [5,7,8] and in Inuit living on the traditional Greenlandic marine diet [9,10].

The line of adverse effects of POPs include endocrine disruption within the reproductive system, central nervous system with developmental and behavioral disabilities, cancer, metabolic disorders, the immune system and cardiovascular disease [1,2,11]. A main background for cardiovascular disease (CVD) is atherosclerosis, and chronic inflammation has been suggested to be involved in development of atherosclerosis and thus CVD [12]. Previously, we found high levels of markers of inflammation with a frequent intake of the traditional Inuit diet [13]. This diet contains POPs that have been shown to act in a pro-inflammatory manner [11,14]. POPs may thus contribute to the rise in the occurrence of CVD in Greenland [15].

This led us to investigate the association between exposure to POPs from the marine diet and inflammation, among Inuit and non-Inuit living in Greenland, taking into account other factors including vitamin D.

## Subjects and methods

A detailed description of the area of investigation and the subjects in the study has been presented previously [13,16,17]. In short, 535 Inuit and non-Inuit, men and women, 50–69 years old participated. Data were collected in 1998 and participants were living in the capital city Nuuk at the West coast or in rural Ammassalik district at the Greenlandic East coast at the time of the survey.

### Dietary habits

A food frequency questionnaire (FFQ) was used to obtain information regarding dietary habits. It included both traditional Greenlandic food items (seal, whale, wild birds, fish, reindeer, musk ox, hare and lamb) and imported food items (pre-cooked meals, potatoes, vegetables, butter, cheese, eggs and fresh fruit). The participants were asked to categorize the intake of each individual food items ranging from never to daily intake. A frequency score for each food item was then calculated as the average number of days per month it was ingested (daily intake = 30.4; 4–6 times/week = 21.7; 1–3 times/week = 8.7; 2–3 times/month = 2.5; 1 time/month = 1 and never = 0 days/month) [18]. The frequency scores were summed for all food items ingested by each participant: Greenlandic food items were scored positively and imported food items were scored negatively. Participants were then categorized into five diet groups: Diet group 1: >80%; 2:60–79%; 3:40–59%; 4:20–39%; 5: <20% Inuit food item scores on a scale where 100% represented Inuit food scores only and 0% represented imported foods only [16].

### Assays

#### 25-hydroxyvitamin D

25-hydroxyvitamin D3 and D2 were analysed by isotope dilution liquid chromatography-tandem mass spectrometry (LC-MS/MS) and calibrated to the NIST standard, as described in details previously [19].

#### Markers of inflammation

Serum YKL-40 levels were measured with an ELISA method (Quidel), with a measuring range of the assay of 20–300 ng/ml. Intra- and inter-assay CV were 5.8 and 6.0%, respectively. Limit of detection was 5.4ng/ml (information supplied by manufacturer). We measured hsCRP levels with a highly sensitive, latex particle-enhanced immunoturbidimetric assay (DAKO, Glostrup, Denmark), with a measuring range of the assay of 0.2–80 mg/l and detection limit of 0.03 mg/l.

#### Persistent organic pollutants

Samples were analysed for 35 lipophilic POPs at Le Centre de Toxicologie du Québec (Sainte-Foy, Québec, Canada) which is an international certified laboratory established by the Canadian Association for Environmental Analytic Laboratories. Fourteen polychlorinated biphenyls (PCBs) [PCB28, 52, 99, 101, 105, 118, 128, 138, 153, 156, 170, 180, 183, 187], ten flame retardants: one polybrominated biphenyl [PBB153] and nine polybrominated diphenyl ethers (PBDEs) [PBDE15, 17, 25, 28, 33, 47, 99, 100, 153], and eleven organochlorine pesticides (OCPs) [*p*,*p’*-DDT (dichlorodiphenyltrichloroethane) and its major metabolite *p*,*p’*-DDE (dichlorodiphenyldichloroethylene), aldrin, mirex, β-hexachlorocyclohexane (β-HCH), hexachlorobenzene (HCB), cis- and trans-nonachlor, α-, γ- and oxy-chlordane] were analysed in purified extracts by high-resolution gas chromatography with electron capture detection. As POPs are lipophilic, all determined POPs were adjusted to the serum lipid content analysed from the corresponding sample and reported as μg/kg serum lipid. The limit of detection (LOD) was adjusted in function of the lipids for each sample and values below the LOD were imputed to LOD/2.

Blood samples were drawn from the antecubital vein in non-fasting participants using minimal tourniquet. Serum was separated and stored at -20^°^C until analysis. Blood samples for all assays were analysed in random order with participant characteristics blinded to the laboratories.

The survey was conducted in accordance by the guidelines laid down by the Declaration of Helsinki and the procedures were approved by the Commission for Scientific Research in Greenland (jr. number 2015–15701).

All subjects gave informed written consent which was in Greenlandic or Danish as chosen by the participant.

### Statistical analysis

Results are given as number of participants in groups, percentages, medians and interquartile range. Groups were compared using the chi-squared test, Mann-Whitney *U* test for comparison of levels between two groups and Kruskal-Wallis test for levels between several groups. Kendall’s tau rank correlation coefficient was used to describe the trend between several groups. Linear regression analyses were performed to test the correlation between POPs and markers of inflammation. YKL-40 and hsCRP were dependent variables. Firstly, univariate regression was done with ethnicity (Inuit vs non-Inuit), age (continuous, year), gender (men vs women), BMI (continuous, kg/m^2^), smoking (never, past, <11, 11–20, >20 cigarettes per day), alcohol intake (never, 0–7, 8–14, 15–21, >21 units/week), diet (five diet groups), vitamin D (continuous) and POPs (continuous) as explanatory variables. Multiple linear regression analyses were performed to examine the association between the independent variables POPs and the depending variables YKL-40 and hsCRP. Covariates adjusted for in the multiple linear regression models were age, alcohol intake, smoking, BMI and vitamin D, included similarly to the univariate analysis. YKL-40 and hsCRP were logarithmically transformed for the linear regression analyses using the natural logarithm.

MedStat software (Astra) was used for the random selection of participants in Nuuk. Data was processed and analysed using Stata version 13.1 software (StataCorp.). A two-sided p-value less than 0.05 was considered statistically significant.

## Results

Characteristics of the study population based on dietary habits are depicted in Table 1. Intake of a diet consisting mainly of traditional Inuit food items was seen especially among Inuit and among participants of mixed ethnicity. Also, participants in the oldest age group, inhabitants in town or in settlement, smokers and hunters had a higher intake of mainly Greenlandic foods.

**Table 1:** Characteristics of the participants in the survey, based on intake of Greenlandic diet.

				Greenlandic diet			
		80%+	60–79%	40–59%	20–39%	<20%	
		n (%)	n (%)	n (%)	n (%)	n (%)	p^a^
Ethnicity	Inuit	239 (55)	104 (24)	66 (15)	20 (5)	5 (1)	
	Mix	2 (29)	3 (43)	1 (14)	1 (14)	0 (0)	
	Non-Inuit	0 (0)	3 (3)	13 (14)	40 (43)	38 (40)	<0.001
Age	50–59 years	129 (37)	73 (21)	54 (16)	51 (15)	37 (11)	
	60–69 years	112 (59)	37 (19)	26 (14)	10 (5)	6 (3)	<0.001
Gender	Men	139 (45)	54 (17)	42 (14)	42 (14)	32 (10)	
	Women	102 (45)	56 (25)	38 (17)	19 (8)	11 (5)	0.015
Residence	City	44 (21)	48 (23)	50 (24)	45 (21)	24 (11)	
	Town	93 (54)	30 (17)	21 (12)	13 (8)	16 (9)	
	Settlement	104 (69)	32 (21)	9 (6)	3 (2)	3 (2)	<0.001
BMI^b^	Normal	117 (48)	50 (21)	30 (12)	28 (12)	17 (7)	
	Overweight	63 (44)	22 (15)	21 (15)	23 (16)	15 (10)	
	Obese	37 (41)	20 (22)	16 (18)	8 (9)	9 (10)	0.42
Smoking	Never	22 (27)	19 (23)	14 (17)	15 (18)	12 (15)	
	Past	27 (40)	14 (21)	13 (19)	11 (17)	2 (3)	
	Present	191 (50)	77 (20)	53 (14)	35 (9)	29 (7)	0.002
Alcohol^c^	0–7	150 (45)	66 (20)	56 (17)	36 (11)	22 (7)	
	0–14	50 (42)	27 (23)	16 (13)	16 (13)	11 (9)	
	15+	37 (49)	16 (21)	7 (9)	7 (9)	9 (12)	0.569
Hunting	Trade	52 (84)	7 (11)	3 (5)	0 (0)	0 (0)	
	Leisure	92 (45)	49 (24)	25 (13)	23 (11)	14 (7)	
	Never	92 (35)	54 (21)	50 (19)	38 (14)	29 (11)	<0.001

^a^ Compared using chi-squared test.

^b^ Normal corresponds to a BMI between 18.50 and 24.99 kg/m2; overweight corresponds to a BMI between 25 and 29.99 kg/m2 and obese corresponds to a BMI above 30 kg/m2.

^c^ Units per week, one unit equals 8g alcohol.

All samples had levels of OCPs above the detection limit except for aldrin and γ-chlordane. PCB levels were above detection limit in almost all samples but only 5.3% of the samples had PCB52 levels above detection limit. The levels of PBDEs were low. Thus, PBDEs 15, 17, 25 and 33 were not measured above detection limit in any sample, and PBDE99 and PBDE100 had levels above detection limit in less than 10% of the samples (data not shown).

ΣOCP, ΣPCB, ΣPBDE and ΣOCP + ΣPCB are presented in Table 2. The three OCPs (DDE, trans-nonachlor and oxy-chlordane) and the three PCBs (PCB138, PCB153 and PCB180) with the highest measured median levels are presented in Tables 3 and 4. Markedly higher levels of POPs were found in Inuit compared to non-Inuit, with advancing age, among participants living in settlements compared to the capital city of Nuuk, and in participants with a high intake of Greenlandic food items and among those with a frequent main meal from own catch (Tables 2, 3 and 4). In addition, POPs differed with BMI with a general tendency to a higher level in participants with normal BMI (Tables 2, 3 and 4). Higher POP levels were found among present smokers compared to past smokers or those who never smoked, except for DDE. No difference in POP levels were seen with the intake of alcohol.

**Table 2:** Serum concentrations (μg/kg lipid) of summed organochlorine pesticides (ΣOCP), summed polychlorinated biphenyls ΣPCB), summed polybrominated diphenyl ethers (ΣPBDE) and summed OCP + summed PCB (ΣOCP+ΣPCB) among participants in the study.

		ΣOCP			ΣPCB			ΣPBDE			ΣOCP+ΣPCB		
		Median (25;75)	p^a^	p^b^	Median (25;75)	p^a^	p^b^	Median (25;75)	p^a^	p^b^	Median (25;75)	p^a^	p^b^
All participants		5192 (2535;8043)			5303 (1983;8701)			32 (23;44)			10669 (4207;16786)		
Ethnicity	Non-Inuit	498 (291;834)			609 (462;849)			21 (19.3;25)			1094 (754;1673)		
	Inuit	5912 (4074;8525)	<0.001		6567 (4055;9501)	<0.001		35 (26;47)	<0.001		12688 (8105;18283)	<0.001	
Age	50–59 years	4612 (1683;7653)			4732 (1202;8028)			31 (22;43)			9619 (2954;15439)		
	60–69 years	5893 (3785;8751)	<0.001		6801 (3421;10258)	<0.001		33 (24;47)	0.002		12870 (6901;19216)	<0.001	
Gender	Men	5099 (1611;7825)			5507 (1438;8878)			34 (23;47)			11045 (3032;16532)		
	Women	5246 (2917;8154)	Ns		5257 (2446;8639)	ns		31 (23;39)	0.012		10392 (5565;16936)	ns	
Residence	City	2851 (1028;4753)			2402 (904;4120)			24 (20;32)			5340 (1925;8520)		
	Town	5654 (3578;8226)			6482 (3966;8795)			32 (24;43)			12111 (7495;16969)		
	Settlement	7721 (5668;10193)	<0.001	<0.001	9090 (6919;12375)	<0.001	<0.001	46 (34;59)	<0.001	<0.001	16799 (13052;22474)	<0.001	<0.001
BMI^c^	Normal	5649 (3292;8348)			6597 (3154;9509)			38 (26;48)			12488 (6962;17686)		
	Overweight	4386 (1067;7267)			4398 (954;7790)			29 (22;43)			8643 (2054;14637)		
	Obese	5297 (2634;8692)	0.020	ns	5017 (2051;7886)	0.001	0.002	29 (21;38)	<0.001	<0.001	10234 (4329;16049)	0.006	0.024
Smoking	Never	4077 (1001;7334)			2706 (895;7568)			27 (20;38)			6708 (1846;15774)		
	Past	4496 (1155;7854)			4168 (1015;7886)			26 (21;40)			8853 (2170;15232)		
	Present	5402 (3055;8092)	0.032	0.009	6038 (2821;9011)	<0.001	<0.001	33 (24;46)	<0.001	<0.001	11528 (6041;16936)	0.004	<0.001
Alcohol^d^	0–7	5334 (2598;8422)			5303 (2051;8970)			32 (24;44)			10697 (4668;17167)		
	0–14	4887 (1961;7802)			5043 (1669;8465)			33 (23;47)			10191 (3628;15161)		
	15+	4974 (2229;7403)	Ns	ns	5857 (1855;8638)	ns	ns	33 (23;44)	ns	ns	11108 (3735;16348)	ns	ns
Greenlandic diet	80%+	7322 (5268;9719)			8010 (5868;10805)			42 (32;54)			15330 (11523;20266)		
	60–79%	5342 (3331;7663)			5277 (3775;8264)			31 (25;40)			10853 (7423;16348)		
	40–59%	3743 (1955;4959)			2926 (1555;4907)			24 (21;32)			6897 (3628;9552)		
	20–39%	853 (494;1728)			849 (599;1316)			23 (19.5;24)			1731 (1088;3175)		
	<20%	402 (271;784)	<0.001	<0.001	523 (462;776)	<0.001	<0.001	20 (18.5;25)	<0.001	<0.001	967 (746;1440)	<0.001	<0.001
Diet from own catch	Weekly	6521 (4802;9172)			7390 (5283;10265)			38 (31;49)			14306 (10431;19289)		
	Monthly	5253 (2465;7927)			5314 (1865;8204)			32 (24;43)			10762 (4201;16597)		
	Rarely	2750 (711;6063)	<0.001	<0.001	2227 (697;5257)	<0.001	<0.001	24 (20;37)	<0.001	<0.001	5252 (1440;10780)	<0.001	<0.001

ns: p>0.10.

^a^ Groups were compared using Mann-Whitney U test for comparison of levels between two groups and Kruskal-Wallis test for levels between several groups.

^b^ Kendall's tau coefficient for trend.

^c^ Normal corresponds to a BMI between 18.50 and 24.99 kg/m2; overweight corresponds to a BMI between 25 and 29.99 kg/m2 and obese corresponds to a BMI above 30 kg/m2.

^d^ Units per week, one unit equals 8g alcohol.

**Table 3:** Serum concentrations (μg/kg lipid) of selected organochlorine pesticides (OCPs) among participants in the study.

		*p*,*p'*-DDE			Trans-nonachlor			Oxy-chlordane		
		Median (25;75)	p^a^	p^b^	Median (25;75)	p^a^	p^b^	Median (25;75)	p^a^	p^b^
All participants		2300 (1100;3800)			1100 (430;1600)			650 (220;1100)		
Ethnicity	Non-Inuit	350 (180;580)			25 (15;46)			14 (9.6;22)		
	Inuit	2700 (1800;4300)	<0.001		1300 (845;1800)	<0.001		795 (450;1200)	<0.001	
Age	50–59 years	2100 (890;3500)			950 (230;1500)			565 (86;1000)		
	60–69 years	2700 (1700;4200)	<0.001		1300 (770;1800)	<0.001		780 (370;1200)	<0.001	
Gender	Men	2300 (865;3600)			1100 (260;1600)			675 (93;1100)		
	Women	2300 (1400;4200)	0.048		990 (555;1600)	ns		640 (270;1100)	ns	
Residence	City	1400 (630;2400)			550 (85;970)			260 (44;460)		
	Town	2600 (2300;5100)			1250 (730;1700)			790 (410;1100)		
	Settlement	3300 (2300;5100)	<0.001	<0.001	1600 (1100;2000)	<0.001	<0.001	1200 (820;1500)	<0.001	<0.001
BMI^c^	Normal	2500 (1400;3750)			1200 (660;1700)			805 (350;1200)		
	Overweight	2100 (645;3400)			880 (130;1500)			485 (54;960)		
	Obese	2600 (1400;4600)	0.031	ns	980 (440;1600)	0.004	0.008	550 (220;1000)	<0.001	0.001
Smoking	Never	2100 (630;4200)			750 (54;1300)			350 (28;900)		
	Past	2200 (650;3700)			900 (160;1500)			530 (73;1100)		
	Present	2300 (1400;3700)	ns	ns	1100 (620;1700)	<0.001	<0.001	720 (300;1100)	<0.001	<0.001
Alcohol^d^	0–7	2400 (1200;3900)			1100 (490;1700)			680 (240;1100)		
	0–14	2050 (1100;3400)			995 (400;1600)			585 (175;1100)		
	15+	2100 (1200;3700)	ns	ns	1100 (325;1500)	ns	ns	700 (145;1000)	ns	ns
Greenlandic diet	80%+	3200 (2300;4800)			1500 (1100;2000)			1000 (720;1400)		
	60–79%	2400 (1600;3900)			1100 (730;1500)			675 (360;970)		
	40–59%	1800 (1000;2900)			700 (330;1000)			330 (140;600)		
	20–39%	570 (330;960)			50 (25;220)			25 (14;91)		
	<20%	260 (180;580)	<0.001	<0.001	19 (13;37)	<0.001	<0.001	11 (8.5;22)	<0.001	<0.001
Diet from own catch	Weekly	3100 (2100;4600)			1400 (990;1900)			910 (630;1300)		
	Monthly	2200 (1200;3650)			1000 (405;1600)			620 (180;1100)		
	Rarely	1200 (520;2700)	<0.001	<0.001	570 (36;1250)	<0.001	<0.001	260 (21;705)	<0.001	<0.001

ns: p>0.10.

^a^ Groups were compared using Mann-Whitney U test for comparison of levels between two groups and Kruskal-Wallis test for levelsbetween several groups.

^b^ Kendall's tau coefficient for trend.

^c^ Normal corresponds to a BMI between 18.50 and 24.99 kg/m2; overweight corresponds to a BMI between 25 and 29.99 kg/m2 and obese corresponds to a BMI above 30 kg/m2.

^d^ Units per week, one unit equals 8g alcohol.

**Table 4:** Serum concentrations (μg/kg lipid) of selected polychlorinated biphenyls (PCBs) among participants in the study.

		PCB138			PCB153			PCB180		
		Median (25;75)	p^a^	p^b^	Median (25;75)	p^a^	p^b^	Median (25;75)	p^a^	p^b^
All participants		620 (280;935)			1800 (620;2800)			1300 (415;2500)		
Ethnicity	Non-Inuit	86 (58;130)			190 (130;250)			140 (110;200)		
	Inuit	730 (490;1000)	<0.001		2100 (1300;3000)	<0.001		1700 (940;2700)	<0.001	
Age	50–59 years	590 (180;880)			1500 (380;2500)			1200 (270;2200)		
	60–69 years	720 (410;1100)	<0.001		2200 (1100;3200)	<0.001		1750 (720;2800)	<0.001	
Gender	Men	610 (195;885)			1750 (415;2800)			1400 (310;2600)		
	Women	640 (340;1000)	0.082		1800 (795;2800)	ns		1300 (465;2300)	ns	
Residence	City	330 (150;530)			770 (280;1400)			480 (200;1000)		
	Town	725 (405;990)			2050 (1250;2850)			1700 (895;2500)		
	Settlement	860 (670;1200)	<0.001	<0.001	2800 (2200;3900)	<0.001	<0.001	2700 (1900;3800)	<0.001	<0.001
BMI^c^	Normal	665 (390;865)			2000 (1000;2950)			1850 (735;2800)		
	Overweight	515 (155;870)			1400 (295;2550)			955 (210;2000)		
	Obese	640 (300;1000)	0.057	ns	1600 (620;2700)	0.006	0.011	1100 (370;1700)	<0.001	<0.001
Smoking	Never	450 (120;970)			910 (270;2500)			470 (180;1700)		
	Past	510 (150;850)			1300 (310;2500)			960 (250;2300)		
	Present	650 (380;940)	0.013	0.003	1900 (910;2800)	0.001	<0.001	1600 (620;2600)	<0.001	<0.001
Alcohol^d^	0–7	630 (290;970)			1800 (650;2800)			1300 (420;2500)		
	0–14	605 (245;895)			1700 (590;2600)			1350 (415;2550)		
	15+	620 (275;890)	ns	ns	1850 (550;2800)	ns	ns	1600 (330;2500)	ns	ns
Greenlandic diet	80%+	860 (620;1100)			2500 (1800;3400)			2300 (1500;3300)		
	60–79%	640 (430;860)			1800 (1100;2700)			1300 (820;2100)		
	40–59%	410 (230;670)			950 (490;1600)			660 (360;1300)		
	20–39%	130 (85;180)			250 (180;390)			180 (130;290)		
	<20%	75 (57;120)	<0.001	<0.001	150 (120;230)	<0.001	<0.001	140 (110;200)	<0.001	<0.001
Diet from own catch	Weekly	820 (590;1100)			2400 (1800;3400)			2200 (1300;2900)		
	Monthly	605 (265;890)			1750 (590;2600)			1300 (370;2300)		
	Rarely	325 (110;695)	<0.001	<0.001	735 (215;1800)	<0.001	<0.001	480 (130;1400)	<0.001	<0.001

ns: p>0.10.

^a^ Groups were compared using Mann-Whitney U test for comparison of levels between two groups and Kruskal-Wallis test for levels between several groups.

^b^ Kendall's tau coefficient for trend.

^c^ Normal corresponds to a BMI between 18.50 and 24.99 kg/m2; overweight corresponds to a BMI between 25 and 29.99 kg/m2 and obese corresponds to a BMI above 30 kg/m2.

^d^ Units per week, one unit equals 8g alcohol.

**Fig 1:**
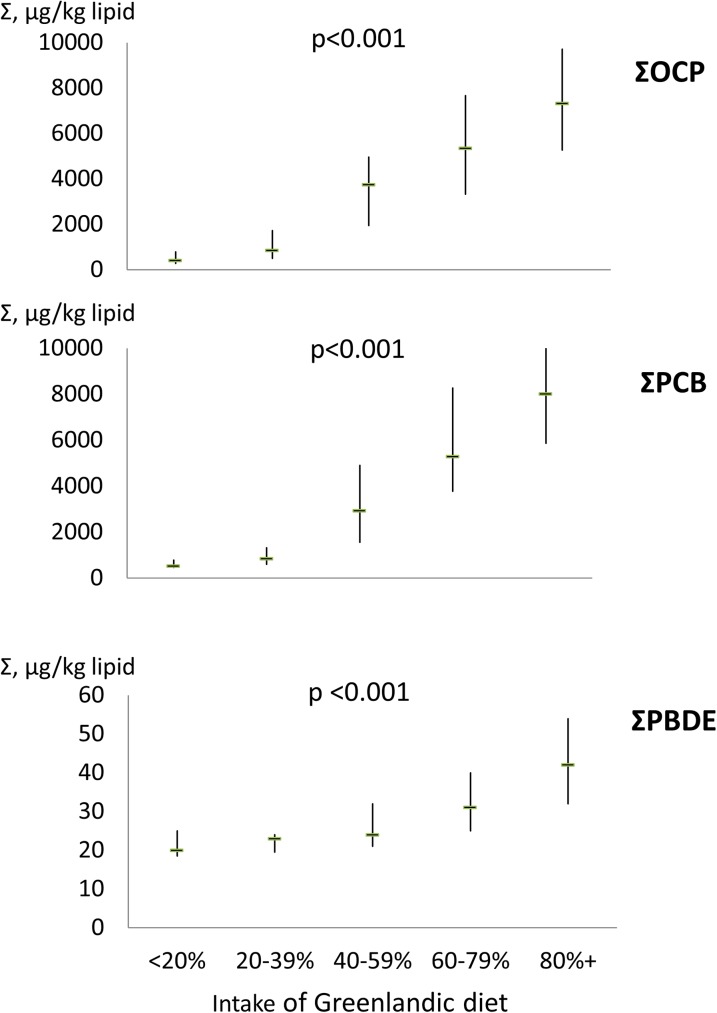
The association between intake of Greenlandic food items and OCPs (top panel), PCBs (middle panel) and PBDEs (bottom panel). Median levels are shown with the 25^th^ and 75^th^ percentiles. P-value is for trend.

Fig 1 illustrates the association between intake of Greenlandic food items and ΣOCP, ΣPCB and ΣPBDE. A statistically highly significant trend was seen with higher levels of all three POP groups with the most frequent intake of Greenlandic food items. As can be seen the median levels rise markedly. Specifically, median level of oxy-chlordane was 91 times higher in individuals with the highest intake of Greenlandic diet compared to those with the lowest intake of Greenlandic food items (Table 3). Trans-nonachlor was 79 times and DDE was 12 times higher. Similarly, median levels of PCB138, PCB153 and PCB180 were 11, 17 and 16 times higher in individuals with highest intake of Greenlandic food items compared to those with an intake of mainly imported food items (Table 4). Despite relatively low levels of PBDEs, median ΣPBDE was two times higher in participants with the highest compared to lowest intake of Greenlandic food items (Table 2).

**Fig 2:**
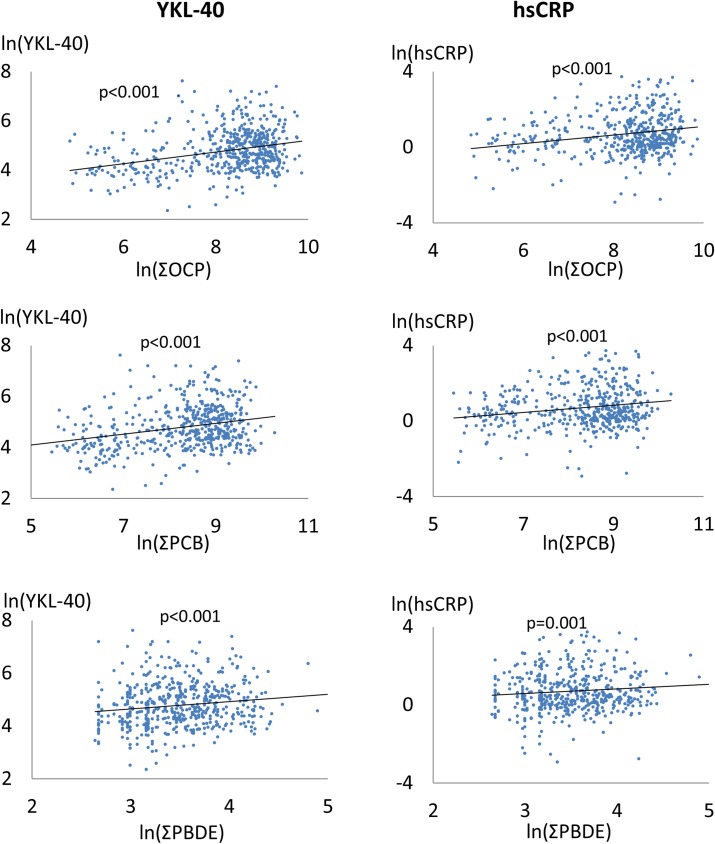
The correlation between the two markers of inflammation and ΣOCP, ΣPCB and ΣPBDE. Linear trendline is shown, p- value is for trend. Ln-transformation solely for presentation.

The correlation between the two markers of inflammation (YKL-40 and hsCRP) and ΣOCP, ΣPCB and ΣPBDE is depicted in Fig 2. A marked trend was seen with increasing levels of both markers of inflammation with higher POP exposure.

Table 5 shows results from univariate linear regression analyses with YKL-40 and hsCRP as dependent variables and ethnicity, age, gender, BMI, smoking, alcohol intake, Greenlandic diet, vitamin D and the selected POPs as individual explanatory variables. Both markers of inflammation were higher in Inuit compared to non-Inuit, increased with age and decreased with decreasing intake of Greenlandic food items. YKL-40 levels decreased with increasing BMI and were higher in smokers. hsCRP levels decreased with an increase in alcohol intake (Table 5). No correlation was found between exposure to PBDEs and either of the two markers of inflammation. Exposure to individual and the sum of OCPs and PCBs associated with a highly significant rise in both markers of inflammation in the crude linear regression analysis (Table 5).

**Table 5:** Factors important to YKL-40 and hsCRP among populations in Greenland participating in the survey in univariate linear regression model.

		YKL-40		hsCRP	
	n	β	P	β	p
Ethnicity^a^	523	0.69	<0.001	0.49	0.001
Age	530	0.04	<0.001	0.03	0.001
Gender^b^	530	0.09	ns	0.03	ns
BMI	500	-0.02	0.002	0.007	ns
Smoking^c^	529	0.07	0.032	-0.02	ns
Alcohol^d^	522	0.04	ns	-0.16	<0.001
Diet^e^	530	-0.18	<0.001	-0.13	0.002
Vitamin D	526	-0.0001	ns	0.002	ns
OCP					
DDE	530	0.0001	<0.001	0.0001	<0.001
Trans-nonachlor	530	0.0002	<0.001	0.0002	<0.001
Oxy-chlordane	530	0.0002	<0.001	0.0003	0.002
ΣOCP	530	0.00005	<0.001	0.0001	<0.001
PCB					
PCB138	530	0.0003	<0.001	0.0003	0.003
PCB153	530	0.0001	<0.001	0.0001	0.004
PCB180	530	0.0001	<0.001	0.0001	0.005
ΣPCB	522	0.00004	<0.001	0.00003	0.003
ΣOCP+ΣPCB	522	0.00002	<0.001	0.00002	<0.001
PBDE					
ΣPBDE	528	0.002	ns	0.001	ns

p>0.10:ns.

^a^ Inuit vs non-Inuit (non-Inuit reference).

^b^ Men reference.

^c^ Cigarettes per day: never (reference), past, <11, 11–20, >20.

^d^ Units per week: never (reference), 0–7, 8–14, 15–21, >21.

^e^ Diet by five groups (Intake of Greenlandic food items reference).

In the multivariate analysis the same pattern was seen after adjustment for age, alcohol intake, smoking, BMI and vitamin D: no association between exposure to PBDEs and markers of inflammation, but a marked influence on both markers of inflammation by exposure to OCPs, PCBs or the sum of OCPs and PCBs (Table 6).

**Table 6:** Multivariate linear regression adjusting for age, alcohol intake, smoking, BMI and vitamin D on each POP individually or the sum of OCP, PCB, OCP+PCB and PBDE.

		YKL-40		hsCRP	
	N	β	p	β	p
OCP					
DDE	488	0.0001	<0.001	0.0001	<0.001
Trans-nonachlor	488	0.0002	<0.001	0.0002	0.001
Oxy-chlordane	488	0.0002	<0.001	0.0003	0.002
ΣOCP	488	0.00005	<0.001	0.00005	<0.001
PCB					
PCB138	488	0.0004	<0.001	0.0003	0.007
PCB153	488	0.0001	<0.001	0.0001	0.008
PCB180	488	0.0001	0.001	0.0001	0.006
ΣPCB	482	0.00003	<0.001	0.00003	0.006
ΣOCP+ΣPCB	482	0.00002	<0.001	0.00002	0.001
PBDE					
ΣPBDE	486	0.001	ns	0.001	ns

p>0.10:ns.

## Discussion

This is the first population-based study to assess the association between exposure to POPs from the traditional Inuit diet and markers of inflammation in a population in Greenland. We found markedly higher levels of POPs in individuals with the highest intake of traditional Greenlandic food items both in the direct comparisons and after adjusting for other factors known to influence inflammation. Thus, the hypothesised positive association between exposure to POPs from the traditional Inuit diet and markers of inflammation was confirmed.

We evaluated inflammation by measuring serum levels of YKL-40 and hsCRP, which are biomarkers of inflammation [12,20]. The two markers differ in that YKL-40 is secreted by various activated cell-types including macrophages and vascular smooth muscle cells in the vessel walls while CRP is produced in hepatocytes in response to interleukins [12]. We thus chose two different paths involved in the inflammatory process.

The inflammatory process may play a role in the development of atherosclerosis and hence in the development of ischemic heart disease (IHD) [12]. The suggested low occurrence of IHD among pre-western Inuit in Greenland was confirmed recently through evaluation of historic data [21]. It was speculated to be due to the high intake of marine food items rich in n-3 fatty acids [6]. However, a marked increase in IHD has occurred [15]. This could relate to the emergence of a sedentary lifestyle, but IHD occurs also in more remote areas of Greenland where inhabitants have a more active life style [15]. Thus, other factors are likely to contribute. One such factor could be inflammation related to the intake of contaminants from the marine diet [13].

Concentrations of POPs are high in fish and marine mammals [5,7] as well as in Inuit living mainly on traditional marine food items [9]. We confirmed these results as we found significantly higher levels of POPs among participants with a high intake of Greenlandic food items and among those with a weekly diet from own catch compared to low intake groups (Tables 2, 3 and 4). Among the PCBs, PCB138, 153 and 180 had the highest serum levels measured, and among the OCPs, DDE, trans-nonachlor and oxy-chlordane had the highest serum levels measured. These findings are in keeping with other studies from Greenland [9].

The high levels of OCPs and PCBs correlated positively with both markers of inflammation in Inuit, in the crude comparison as well as in the adjusted analysis. This extends previous *in vitro* studies. Hence, Kim *et al*. [22] showed that POPs can regulate genes involved in the inflammatory response of human adipocyte stem cells and thereby contribute to inflammation. Hong *et al*. [23] examined the effect of fish oil contaminated with OCPs and PCBs on inflammation in rats. They found the anti-inflammatory effects of fish oil not to be compromised by the presence of contaminants. However, they included only CRP as a measure of inflammation. Our study extends these findings by investigating both long-term, high-level exposure of POPs and the influence in humans. Hence, the results from animal studies might not easily compare to humane studies.

POPs have been shown to modify the immune response in humans [1,2,11]. Thus, exposure to PCBs associated with increased cytokine levels in Canadian First Nations Communities [24] and exposures to OCPs were associated with increased CRP among non-diabetic adults [25]. The findings by Kumar et al [26] suggest that exposure to POPs is associated with higher levels of some but not all tested inflammatory markers. The potential immune-toxicological effect of POPs were demonstrated in a study by Heilmann *et al*., where a reduced antibody response to vaccinations was seen among children with increased perinatal exposure to PCBs [27]. Thus, there is indirect evidence of a pro-inflammatory as well as an immune-toxicological effect of exposure to POPs. The suggested link between inflammation and POPs is strengthened by our findings of increased levels of markers of inflammation in individuals with high levels of POPs. Hence, a pro-inflammatory role of POPs is plausible and it is further supported by our data.

Participants living on the traditional Greenlandic diet had markedly higher levels of all POPs. The traditional Greenlandic diet consists mainly of fish and marine mammals [6] and it thus becomes the major route of exposure to POPs among the population in Greenland. This was confirmed by our data of higher levels in Inuit compared to non-Inuit, in participants living in settlements, and in those with a weekly intake of diet from own catches (Tables 2, 3 and 4). Covariance exists between these variables as Inuit may rely more on traditional food items and more frequently live in settlements and go hunting compared to non-Inuit. Thus, these are three different measures to support the importance of the diet for POP exposure among populations in Greenland.

Adipose tissue mass is a storage compartment for lipophilic POPs [28]. It associates with BMI. BMI influenced the concentration of POPs in our study. The association between BMI and POPs was non-linear with the lowest level in the overweight participants. POP levels were adjusted to the serum lipid content. This could be speculated to contribute to the non-linear association between BMI and POP levels with higher POP levels among obese participants. The higher POP levels with normal BMI could be due to less storage capacity in this group of participants.

We included 35 different POPs. The three OCPs and PCBs with the highest levels were used for the linear regression analysis, and the sum of PCBs, OCPs and PBDEs as well as sum of PCBs+OCPs were also included in the linear regression analysis. These models thus included all measured POPs in the assessments.

Mercury, lead and cadmium were not included in the measurements. Marine mammals are the main source of exposure to mercury in the Arctic and mercury concentrations are high in marine mammals [29]. However, smoking is also a route of exposure to mercury as well as to lead and cadmium. These metals may also act in a pro-inflammatory way and increase oxidation [30]. We adjusted for smoking in the multiple linear regression models. Hence, the association between POPs and markers of inflammation has indirectly been adjusted for at least some of the influence of these metals.

Inflammatory markers may be influenced by factors not accounted for in this study such as physical activity. A further limitation is the cross-sectional design, which is a shortcoming in the consideration of causality. However, the influence of intake of traditional diet on inflammation can hardly be studied in a non-observational design. Our population-based study included one percent of the population of Greenland and the participation rate was 95%. We surveyed two areas in Greenland selected to represent the diversity in life style in Greenland from settlements in rural East Greenland to the capital city Nuuk in West Greenland. This wide range in geography and lifestyle demonstrated marked differences in exposure to the POPs related to differences in dietary habits. The validity of dietary assessment by the FFQ was strengthened by crosscheck questions and a biomarker of the marine diet as described previously [16,31].

Participants in the older age group had significantly higher levels of POPs in keeping with previous studies [4,8,10]. Elderly people tend to live more traditionally than do the younger generations and they have a higher intake of traditional Greenlandic food items. Also, the only routes of excretion of POPs are trans-placental from mother to foetus and through breastfeeding [32,33]. Hence, POPs accumulate in the body over time leading to higher levels with increasing age [8,10]. Thus, the load of POPs is determined by individual dietary intake and duration of exposure. We measured POPs at one point in time due to the cross-sectional nature of the study. However, the participants were in an age group, where changes in lifestyle and diet had been limited. Hence, the dietary habits reported in the FFQ are likely to reflect the load of exposure to POPs over time.

The traditional pre-western Inuit diet was rich in protein and fat, including the n-3 poly-unsaturated fatty acids, while low in carbohydrates. Thus, the composition differs markedly from that of the imported diet [6]. The Inuit diet contains high levels of vitamin D [17,31] and other nutrients such as iodine, selenium and vitamin E [16,34]. However, it has become evident that also the levels of contaminants are high [7]. Hence, it provides both nutrients and contaminants in addition to adding to the Inuit identity, and this delicate balance has been labelled ‘The Arctic dilemma’ [7]. While the levels of some contaminants are declining over time, others are increasing [7,9], and the traditional Inuit diet is a major source of exposure to contaminants even today. This makes dietary recommendations for guidance of Greenland Inuit [35] towards a healthy lifestyle complex and our results support guidance based on knowledge rather than notions.

In conclusion, we found markedly higher levels of POPs in individuals in Greenland with a traditional way of living, and POPs were associated with the intake of the traditional Inuit diet. Interestingly, POPs correlated positively with markers of inflammation. The latter is in keeping with studies suggesting a pro-inflammatory role of POPs. Inflammation plays a role in the development of a number of chronic diseases such as rheumatoid arthritis and atherosclerosis and hence cardiovascular disease. These are common diseases in populations in Greenland and our results add to the knowledge on the consequences of POPs in exposed individuals. These data may also inform guidelines on ‘the Arctic dilemma’, and they encourage follow-up on the ageing Arctic populations.
